# Mass Spectrometry Imaging and Identification of Peptides Associated with Cephalic Ganglia Regeneration in Schmidtea mediterranea*[Fn FN2]

**DOI:** 10.1074/jbc.M115.709196

**Published:** 2016-02-16

**Authors:** Ta-Hsuan Ong, Elena V. Romanova, Rachel H. Roberts-Galbraith, Ning Yang, Tyler A. Zimmerman, James J. Collins, Ji Eun Lee, Neil L. Kelleher, Phillip A. Newmark, Jonathan V. Sweedler

**Affiliations:** From the ‡ Department of Chemistry, and the Beckman Institute,; the § Department of Cell and Developmental Biology, Howard Hughes Medical Institute, University of Illinois at Urbana-Champaign, Urbana, Illinois 61801, and; the ¶ Departments of Chemistry and Molecular Biosciences, Proteomics Center of Excellence, Northwestern University, Evanston, Illinois 60611

**Keywords:** imaging, mass spectrometry (MS), neurogenesis, neuropeptide, regeneration

## Abstract

Tissue regeneration is a complex process that involves a mosaic of molecules that vary spatially and temporally. Insights into the chemical signaling underlying this process can be achieved with a multiplex and untargeted chemical imaging method such as mass spectrometry imaging (MSI), which can enable *de novo* studies of nervous system regeneration. A combination of MSI and multivariate statistics was used to differentiate peptide dynamics in the freshwater planarian flatworm *Schmidtea mediterranea* at different time points during cephalic ganglia regeneration. A protocol was developed to make *S. mediterranea* tissues amenable for MSI. MS ion images of planarian tissue sections allow changes in peptides and unknown compounds to be followed as a function of cephalic ganglia regeneration. In conjunction with fluorescence imaging, our results suggest that even though the cephalic ganglia structure is visible after 6 days of regeneration, the original chemical composition of these regenerated structures is regained only after 12 days. Differences were observed in many peptides, such as those derived from secreted peptide 4 and EYE53-1. Peptidomic analysis further identified multiple peptides from various known prohormones, histone proteins, and DNA- and RNA-binding proteins as being associated with the regeneration process. Mass spectrometry data also facilitated the identification of a new prohormone, which we have named secreted peptide prohormone 20 (SPP-20), and is up-regulated during regeneration in planarians.

## Introduction

Understanding mechanisms of tissue regeneration is significant for biomedical research because many diseases, in particular neurodegenerative diseases that affect the CNS, do not have a cure. A simple organism like the freshwater planarian *Schmidtea mediterranea* is an ideal model for studying the molecular mechanisms that guide the regenerative process because these freshwater flatworms can regenerate missing tissue (1, 2). The CNS of *S. mediterranea* is characterized by bi-lobed anterior cephalic ganglia with two nerve cords that run along the ventral surface of the animal. When the head is amputated, a planarian will regenerate the ganglia and reconnect them to the remaining ventral nerve cords (3). Stem cells called neoblasts, which are distributed throughout the planarian body, govern structural and functional regeneration of organs and reestablishment of body polarity after significant physical injury (4–10).

Results from past and current studies have produced a wealth of information on the planarian CNS, showing that many fundamental aspects of neurobiology are conserved between planarians and humans (11), *e.g.* the use of signaling molecules such as neuropeptides (12–14). The possible involvement of neurotransmitters, neuropeptides, and antimicrobial peptides in CNS regeneration has been suggested in prior studies (15–20). Biochemical differences between intact and regenerating planarians have been investigated using a variety of genomic, transcriptomic, and proteomic techniques (3, 7, 8, 11, 16, 21–33).

In this work, we contribute information on the chemical, spatial, and temporal changes that take place during the regeneration process by focusing primarily on neuropeptide and protein changes. We employed MALDI MS in an imaging modality (mass spectrometry imaging; MSI)^6^ to acquire chemical data with spatial information (34–36). MS is an untargeted and multiplex analysis technique that has already been used to conduct a quantitative investigation of the neoblast proteome (37). MALDI is well suited for analyzing biological samples because of its large mass range and general tolerance to salts (38). MALDI MS, in a non-imaging application, has been used to study protein expression changes during planarian anterior regeneration (39). In MALDI MSI, as used here, the laser is rastered across the sample to achieve position-resolved MS acquisition (34–36). MALDI MSI has been applied to studies of peptide and protein changes during nervous system regeneration in the medicinal leech, reporting an interaction between the nerve cord and the surrounding blood sinus (40, 41). In our work, we used a similar approach to examine the chemical changes associated with planarian tissue regeneration on both the spatial and temporal levels.

We previously conducted an 'omics investigation of the prohormone complement of the sexual and asexual strains of *S. mediterranea* (23). Here, building on data from that study, we used MALDI MSI to visualize *m*/*z* distribution changes associated with cephalic ganglia regeneration. Detected masses that changed during regeneration were sorted using principal component analysis (PCA), an effective statistical technique for recognizing patterns based on data obtained directly from a tissue sample (42, 43). Finally, we subjected additional sets of planarian samples to LC-MS-based peptidomic analysis to identify the specific peptides involved in regeneration (44–47). To validate the peptide and proteins observed via MS, we conducted *in situ* hybridization, providing mRNA localization of the regeneration markers.

## Experimental Procedures

### 

#### 

##### Chemicals

Chemicals were purchased from Sigma unless noted otherwise.

##### Animal Culture and Cephalic Ganglia Amputation

**FIGURE 1.**
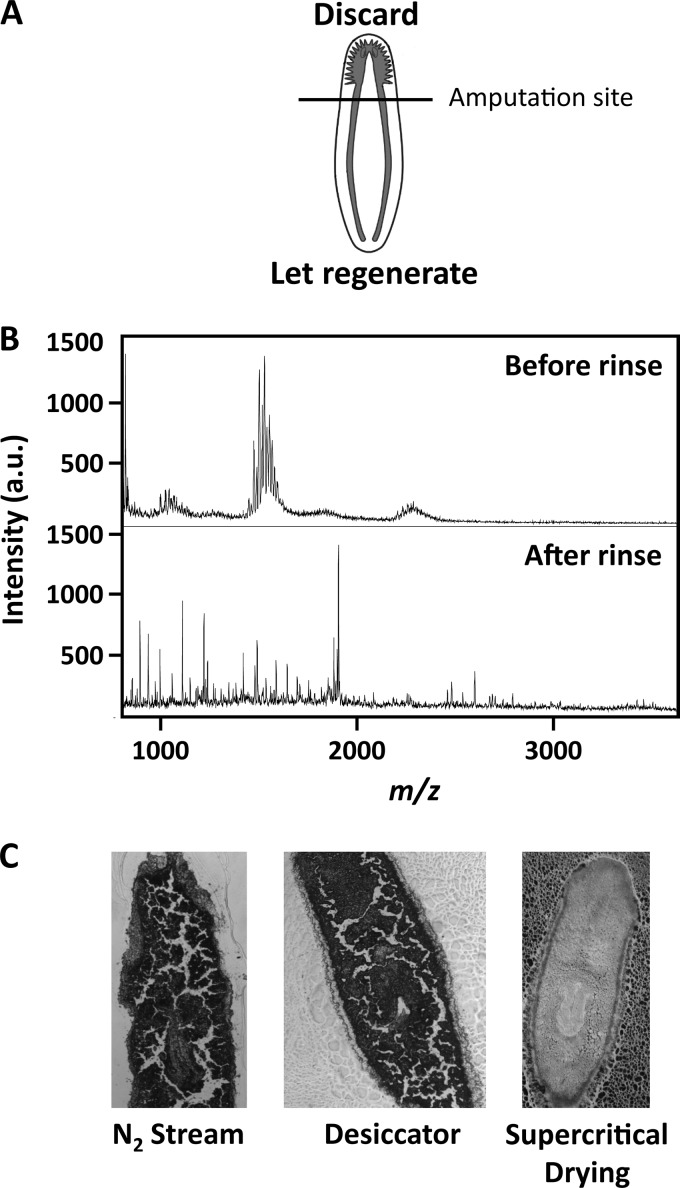
**Experimental protocol for MSI of *S. mediterranea* cephalic ganglia regeneration.** *A*, intact *S. mediterranea* were amputated at the midpoint between the tip of the head and the pharynx. The diagram of the planarian was modified from a previous study (71). *B*, rinsing removes interfering signals at *m*/*z* 1500–1600. *C*, post-rinse drying using typical methods, such as an N_2_ stream or a desiccator, caused tissue sections to crack, whereas supercritical drying preserved tissue section integrity.

Mature, asexual strains of *S. mediterranea*, ∼1 cm in length, were maintained at 20 °C in Instant Ocean salts (Spectrum Brands, Blacksburg, VA) dissolved at 0.5 g/liter in ultrapure water. Animals were starved for at least 1 week prior to analysis to avoid nonspecific background signals from the gut contents (23). To initiate cephalic ganglia regeneration, the planarians were amputated midway between the tip of the head and the pharynx (Fig. 1*A*). The posterior portions of amputated planarians were then returned to the salt solution to regenerate. For the peptidomic analysis of regenerating planarians, 80 planarians were amputated, and the anterior and posterior portions were placed in separate containers of the same salt solution described above.

##### Tissue Embedding and Cryosectioning

Intact planarians, and planarians at 3, 6, or 12 days after amputation, were placed on a Parafilm M substrate and immobilized on ice. Once immobile, each animal was embedded in 10% gelatin and frozen in dry ice-chilled ethanol. Longitudinal sequential tissue sections (20 μm) were taken at −16 °C using a Leica CM 3050 S cryostat (Leica Microsystems, Bannockburn, IL) and individually thaw-mounted onto separate silicon wafers. For tissue sections taken from intact and 12-day regenerated planarians, the cephalic ganglia could be seen under a dissection microscope (Leica MZ75, Leica, Wetzlar, Germany) and were visible among at least one of the four most ventral sections; the sections having visible cephalic ganglia were used for the MSI studies. The cephalic ganglia were not clearly visible under a dissection microscope for sections from 3- and 6-day regenerated planarians. For these two time points, the four most ventral sections were used for MSI so as to be consistent with intact and 12-day regenerated planarians.

##### Tissue Rinsing and Supercritical Drying

Sections to be imaged were rinsed for 10 s in ice-chilled 150 mm ammonium acetate buffer, followed immediately by 100% isopropyl alcohol for 30 s. The rinsed sections were dried using an Autosamdri-931 supercritical dryer (Tousimis, Rockville, MD) with 100% isopropyl alcohol as the intermediate fluid and “bone dry” liquid carbon dioxide (SJ Smith, Davenport, IA) as the supercritical fluid. Supercritical drying was begun by submerging tissue sections in 10 ml of 100% isopropyl alcohol in the supercritical drying chamber. The chamber was then slow filled with liquid carbon dioxide for 90 s before being fully filled at the regular flow rate for 30 s. The isopropyl alcohol was purged from the sample chamber with liquid carbon dioxide for 4 min. After purging, the chamber pressure and temperature were raised and held at above 1200 p.s.i. and 31 °C for 4 min. The chamber was returned to ambient conditions by slowly bleeding the pressure to 550 p.s.i. before fully venting.

##### MALDI Matrix Application

The MALDI matrix α-cyano-4-hydroxycinnamic acid was sublimed onto the sample in a glass sublimation chamber (Ace Glass, Vineland, NJ). The cold finger was chilled using an ice bath, and heating was conducted using a heating mantle powered with a variable autotransformer set to 55% of 120 V (Staco, Dayton, OH). A vacuum was created in the chamber using an Edwards 30 E2M30 rotary pump (Edwards ROC, Sanborn, NY). The setup ran for 18.5 min to create a α-cyano-4-hydroxycinnamic acid coating with a density of ∼0.2 mg/cm^2^. To improve peptide extraction into the matrix, the matrix-coated samples were placed in a humidity chamber with 1 ml of 5% acetic acid for 225 s at 85 °C, following a previously described approach (48).

##### MALDI Mass Spectrometry Imaging

Our protocols for MSI are similar to a prior report (49). Briefly, silicon wafers containing prepared samples were taped to a custom MALDI target using double-sided conductive copper tape (electrical tape, 3M, St. Paul, MN). The target contained a shallow cutout that allowed the wafer surface to be positioned at the same level as the target surface. MSI was conducted in positive mode using an ultrafleXtreme MALDI-TOF/TOF mass spectrometer (Bruker Daltonics, Billerica, MA) equipped with a Smartbeam II frequency tripled Nd:YAG solid state laser. The laser footprint was set to “small” at ∼30 μm diameter. Mass spectra were acquired at 50-μm intervals, and signals from 250 shots fired at 1000 Hz were summed. Images and mass spectra were processed using flexAnalysis and flexImaging software (Bruker Daltonics). The ion images generated in flexImaging were normalized to the total ion count.

##### Nuclear Staining and Fluorescence Imaging

Thaw-mountedplanarian sections were submerged in 1 μg/ml of DAPI for several minutes in ambient conditions and fluorescently imaged using a SteREO Lumar.V12 stereomicroscope (Carl Zeiss, Oberkochen, Germany), equipped with an X-Cite 120 mercury lamp (Excelitas, Ontario, Canada) and a Neolumar S objective lens (1.5×, FWD, 30 mm). Images were captured using an AxioCam MRM camera (Carl Zeiss) and AxioVision software, version 4.6.3 (Carl Zeiss). The acquired images were not processed using any other software.

##### Principal Component Analysis and Determining Regeneration Markers

Given the information-rich nature of MSI data sets, statistical approaches such as PCA can be helpful for reducing the dimensionality of the data set and determining important markers (50). To focus the statistical analysis on the cephalic ganglia and the blastema, these areas were selected as the regions of interest. Regions of interest selection was conducted by generating ion images that showed the ganglia or the blastema, and then manually outlining the relevant structure(s). MALDI mass spectra from each region of interest were converted from the Container format to the XMass format and imported into the ClinProTools software, version 2.2 (Bruker Daltonics), as separate groups based on biological replicates (intact, *n* = 7; 3 days, *n* = 7; 6 days, *n* = 5; 12 days, *n* = 5). Spectra were smoothed using the Savitzky-Golay algorithm for 3 cycles with an *m*/*z* width of 0.1. Baseline subtraction was performed with the Top Hat algorithm with 10% baseline width. Peak picking was performed on the average spectrum from each spectral group representative of a biological replicate, with a signal-to-noise cutoff of 5. For each group, the similarity selection option was used to choose the spectrum closest to the group average for PCA. To determine the ions that varied significantly between regeneration time points, the Anderson-Darling test (PAD) was conducted on each peak to test for a normal distribution. Signals that followed a normal distribution (*P*_PAD_ ≥ 0.05) were tested for significance via analysis of variance. Signals that were not normally distributed (*P*_PAD_ ≤ 0.05) were tested for significance via the Kruskal-Wallis analysis of variance. The Benjamini-Hochberg *p* value adjustment procedure was automatically applied to correct for the multiple hypothesis testing problem commonly associated with MS data (29).

##### Peptide Extraction from Regenerating and Intact Planarians

Peptides were extracted from the anterior and posterior pieces of 6-day regenerated planarians (*n* = 80), including the blastema that developed on both kinds of tissue pieces. Tissues were combined and homogenized in 1 ml of acidified acetone (40:6:1, acetone:water:HCl) with the pH adjusted to 4 using 5 m ammonium hydroxide (23). The homogenate was centrifuged (Eppendorf, Hauppauge, NY) at ∼21,000 × *g* at 4 °C for 15 min. The supernatant was separated from the tissue pellet, dried, and reconstituted in 60 μl of 0.1% formic acid (FA). An oily phase and some undissolved residue were observed after reconstituting in the FA, and these two portions were separately isolated from the primary extract. The tissue pellet, peptide extract, oily phase, and undissolved residue were individually frozen at −80 °C until further processing. Peptides were also extracted and reconstituted in 90 μl of 0.1% FA from a separate batch of intact, non-regenerating planarians (*n* = 58) in a similar manner.

##### First Stage HPLC Fractionation of Tissue Extract

Reconstituted peptide extracts were fractionated using either a Breeze 2 HPLC system (Waters, Milford, MA) equipped with a reversed-phase column (Atlantis T3, C18, 2.1-mm inner diameter × 150 mm length, 5-μm particle size, 100 Å pore size; Waters), or a Magic 2002 analytical LC system (Michrom Bioresources Inc., Auburn, CA), also using a reversed-phase column (Vydac, C18, 2.1-mm inner diameter × 150 mm length, 5-μm particle size, 300 Å pore size; W. R. Grace & Co., Columbia, MD). Solvent gradients were established by mixing solvents A and B. Fractions were collected with an analytical gradient of ∼10–50% solvent B ramped over a period of 35–45 min. For the Breeze 2 HPLC system, solvent A was water + 1% acetonitrile (ACN) + 0.1% FA, and solvent B was ACN + 0.1% FA. For the Magic 2002 analytical LC system, solvent A was 5% ACN + 0.1% FA + 0.01% trifluoroacetic acid (TFA), and solvent B was 95% ACN + 0.1% FA + 0.01% TFA.

##### Solid Phase Extraction

For the extract from regenerating planarians, the oily phase and undissolved residue were each dissolved in 600 μl of 5% methanol and 0.1% FA and desalted via solid phase extraction cartridges (Discovery DSC-18, 1 ml volume, Supelco, Bellefonte, PA) per the manufacturer's directions. Retained peptides were eluted sequentially using 30, 50, and 70% ACN. Eluates were dried and reconstituted in 0.1% FA.

##### HPLC-Electrospray Ionization (ESI) Tandem MS (MS/MS) of Planarian Peptide Extracts

Portions of each first stage fraction from regenerating and intact planarian peptide extracts were taken and evaluated using an ultrafleXtreme MALDI-TOF/TOF mass spectrometer (Bruker Daltonics) to determine peptide content. Fractions with observed peptide signals via MALDI MS were subjected to HPLC-ESI MS/MS analysis using a protocol similar to prior reports (23, 43, 51). These peptide-containing fractions were analyzed using a quadrupole (Q)TOF mass spectrometer (maXis 4G or maXis Impact; Bruker Daltonics) or a Fourier transform (FT)-ion cyclotron resonance (ICR) mass spectrometer (11 Tesla LTQ-FT Ultra or 12 Tesla LTQ-FT Ultra; Thermo Fisher Scientific, Waltham, MA), as described in more detail below. The multiple platforms, one based on the maXis instruments from Bruker (Impact or 4G), and the other based on LTQ-FTMS instruments from Thermo Fisher Scientific (with either 11 tesla or 12 tesla magnets), were used to identify a large number of peptides covering an extended mass range for planarians in two different biological states (intact and regenerating).

First-stage fractions from regenerating planarians were divided into two groups based on the mass of the observed ions in the MALDI spectrum. Fractions containing smaller compounds were analyzed using a QTOF mass spectrometer (maXis 4G or maXis Impact; Bruker Daltonics) coupled to a Dionex UltiMate 3000 nanoLC system (Thermo Fisher Scientific) equipped with a reversed-phase analytical column (Magic C18AQ, C18, 0.1-mm inner diameter × 150 mm length, 3-μm particle size, 200 Å pore size; Michrom). Samples (1–2 μl) were loaded onto a peptide trap column (PepMap100, C18, 300-μm inner diameter × 5 mm length, 5-μm particles, 100 Å pore size, Thermo Fisher Scientific) and desalted with a loading solvent (4% ACN and 0.05% FA) for 5 min before being sent through the analytical column. Separation was conducted at a uniform flow rate of 300 nl/min and a gradient of 20–50% solvent B (80% ACN, 0.05% FA, 0.001% TFA) over 75 min. Solvent A was 0.1% FA. The entire run was 120 min. The MS scan range was set at *m*/*z* 500–3000. The precursor isolation window for collision-induced dissociation was *m*/*z* 8 for precursor ions smaller than *m*/*z* 1000, *m*/*z* 10 for precursor ions between *m*/*z* 1000 and 2000, and *m*/*z* 15 for ions larger than *m*/*z* 2000. Data-dependent precursor selection was used to restrict the scan to three fragmentation spectra for the top three ions per 1 min.

Peptide fractions from regenerating planarians that contained larger compounds were analyzed using an 11T FT-ICR mass spectrometer (LTQ-FT Ultra, Thermo Fisher Scientific) coupled with a nanoLC system (Eksigent 1D plus, AB Sciex, Framingham, MA). The sample (1 μl) was mixed with 9 μl of loading solvent (5% ACN and 0.2% FA), loaded onto a peptide trap column (IntegraFrit Sample Trap, Magic AQ particles, 150-μm inner diameter × 200 mm length, 5-μm particle size, 100 Å pore size, New Objective, Woburn, MA), and desalted for 6 min with the loading solvent. The trap column was then placed in-line with the analytical column (PicoFrit column, Magic AQ particles, 75-μm inner diameter × 150 mm length, 5-μm particle size, 100 Å pore size, New Objective), and the sample eluted over an analytical gradient of 18–45% solvent B (95% ACN and 0.2% FA) over 60 min with a flow rate of 300 nl/min. The entire run was 95 min. Solvent A was 5% ACN and 0.2% FA. The MS acquisition parameters were set to scan at *m/z* 300–2000 with an *m*/*z* 10 precursor isolation window for collision-induced dissociation. Data-dependent precursor selection was restricted to the top three most intense ions. Dynamic exclusion was enabled with a repeat count of 3 and an exclusion duration of 120 s.

In a few trial experiment runs at the beginning of this study, first-stage LC fractions from the intact planarian extract were also analyzed using a similar instrument, a 12T FT-ICR mass spectrometer (LTQ-FT Ultra; Thermo Fisher Scientific) interfaced with a nanoLC system (Eksigent 1D plus, AB Sciex). The sample was loaded with helium bomb pressure (500 p.s.i.) onto a peptide trap column (75-μm inner diameter), 6 cm of which was fritted with LiChrosorb (EM Separations, Gibbstown, NJ) and packed with a C18 solid phase (15 μm, YMC Co., Ltd., Allentown, PA). The sample was separated on an analytical column (ProteoPep IITM, C18, 150 mm length 5-μm particle size, 300 Å pore size, New Objective). Data acquisition consisted of an MS scan at *m*/*z* 500–2000 with an *m*/*z* 10 precursor isolation window for collision-induced dissociation. Dynamic exclusion was enabled with a repeat count of 2, and an exclusion duration of 180 s.

##### Bioinformatic Identification of Planarian Peptides

MS/MS base peak chromatograms from the HPLC-ESI-QTOF analysis were processed using DataAnalysis 4.2 (Bruker Daltonics) for compound spectra selection and charge deconvolution. The fragmentation spectra were exported as Mascot generic files (.mgf) and loaded into PEAKS Studio 7.0 (Bioinformatics Solutions Inc., Waterloo, ON, Canada) for searching against the Uniprot *S. mediterranea* protein database containing 712 entries, which includes all of the previously characterized neuropeptides (23). The HPLC-ESI-FT-ICR MS data set was directly loaded into PEAKS Studio in the .raw format without preprocessing. The following search parameters were used, parent mass error tolerance, 50 ppm for spectra from QTOF and 15 ppm for spectra from FT-ICR MS; fragment mass error tolerance, 0.1 Da; charge options, no correction; enzyme, none; variable post-translational modifications (PTMs), acetylation (K), acetylation (N-term), acetylation (protein N-term), amidation, oxidation, pyro-Glu from Glu, pyro-Glu from Gln, and half of a disulfide bridge; maximum variable post-translational modifications per peptide, 3.

Other than matches against the protein database, *de novo* sequence tags generated by PEAKS Studio were searched against the *S. mediterranea* transcriptome database (52) and BLAST searched against sequences from other species. Sequence tags were first filtered to keep only those with an average local confidence above 80%. Searching was further focused on matching residues having a local confidence higher than 80% to emphasize matching high quality portions of the sequence. For those without BLAST results, SignalP (53) was used to predict if a characteristic signal peptide was present in the sequence.

In addition to using PEAKS Studio, database searching for the HPLC-ESI-FT-ICR MS data set was also conducted using ProsightPC 2.0 (Thermo Fisher Scientific) against the *S. mediterranea* Uniprot protein database (23, 54). Search parameters included an 82-Da precursor tolerance and 25 ppm fragment mass tolerance without protease specificity, and an *E*-value threshold of 1 × 10^−4^.

##### In Situ Hybridization of Regeneration Markers

To investigate planarian gene expression, portions of selected genes were cloned into the pJC53.2 vector and clones were used for riboprobe synthesis as previously described (23). Animals were starved for 1 week before fixation or amputation for regeneration experiments; for *in situ* hybridization experiments, we fixed either uninjured animals or regenerating worms 6 days after head amputation. *In situ* hybridization experiments were performed as reported (55), using an InsituPro VS (Intavis) robot.

## Results

### 

#### 

##### Workflow Optimization for Mass Spectrometry Imaging

Sample preparation is a critical step for MSI experiments, and protocols vary, in part due to tissue properties. In this work, we started by optimizing the protocols to work with planarian tissues. We focused on tissue rinsing steps needed to remove signals that interfere with peptide detection. Multiple rinsing and drying protocols were tested for sample clean-up and for minimizing physical damage to the tissue section. We rinsed with a combination of ammonium acetate buffer and isopropyl alcohol to remove a set of unidentified signals that interfered with peptide detection (Fig. 1*B*). Rinsed tissue sections were then dried with supercritical drying, which minimized cracks from forming in the tissue section as compared with other drying techniques, *e.g.* using an N_2_ stream or a desiccator (Fig. 1*C*).

##### Mass Spectrometry Imaging Reveals Changing Peptide Distributions with Regeneration

**FIGURE 2.**
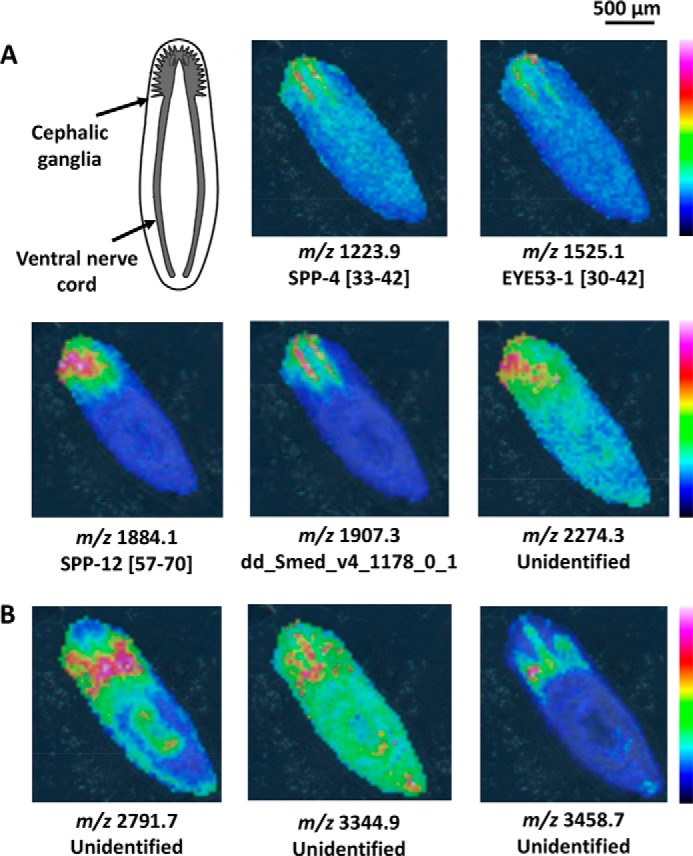
**MALDI MSI ion images for intact planarians.** *A,* many peptides are localized to the cephalic ganglia or toward the anterior end of the planarian. Peptides derived from different SPP are labeled with their sequence portion within the prohormone. Peptides not derived from known prohormones are labeled with their FASTA annotation as entered in the planarian transcriptome. Unidentified ions were detected via MALDI MSI but were not characterized in the follow-up HPLC-ESI MS/MS peptidomic analysis. The diagram of the planarian nervous system shown in the *upper left* was modified from a previous study (71). *B*, peptides that are localized in the mesenchyme around the cephalic ganglia are also observed. The anterior of each animal is positioned toward the *top left*. Signal intensity is color coded, with intensity scales provided. Intensity increases from *blue* to *red*.

**FIGURE 3.**
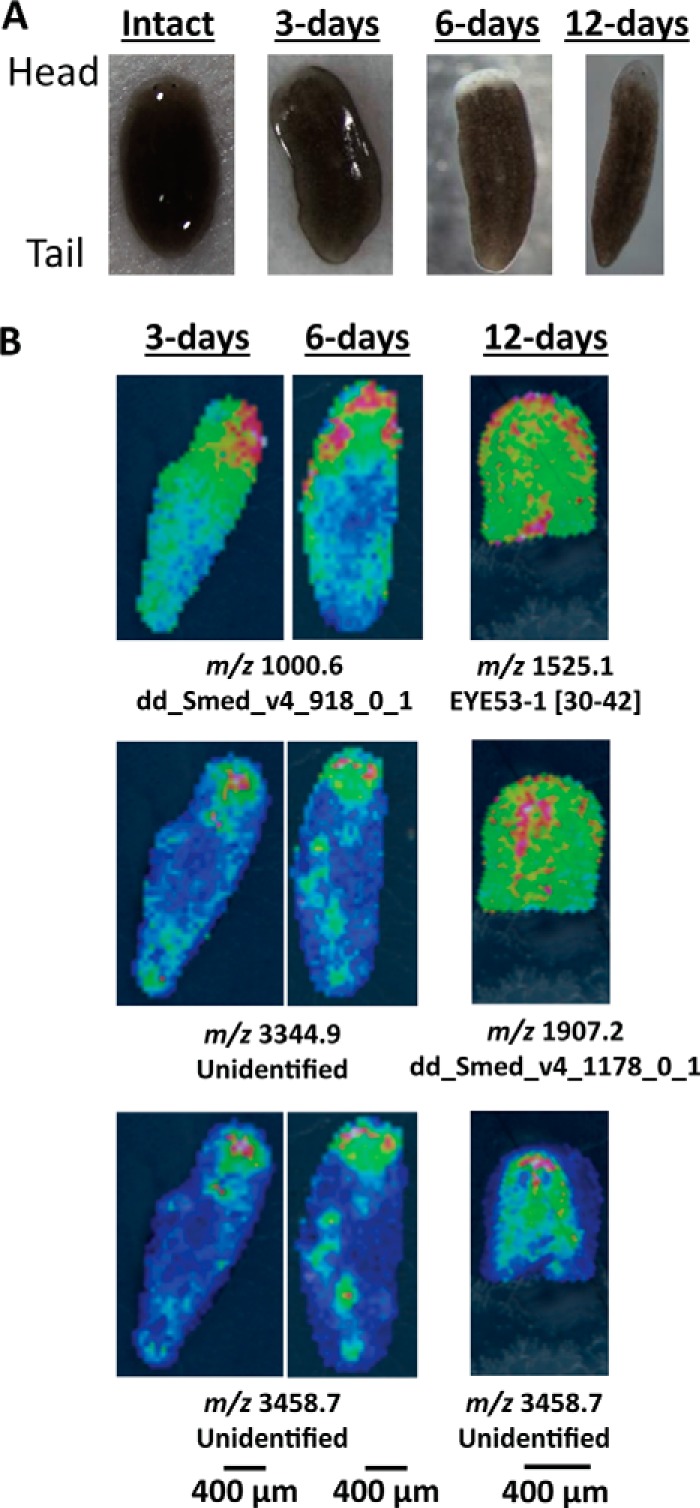
**Optical and ion images for regenerating planarians at 3, 6, and 12 days after amputating the cephalic ganglia.** *A*, optical images of intact and regenerating planarians. *B*, several ions are localized toward the blastema in 3-day and 6-day regenerating planarians. The cephalic ganglia are visible after 12 days of regeneration. In 12-day regenerates, only the anterior half of the planarian is imaged. Peptides derived from known prohormones, such as EYE53-1, are labeled with their sequence portion within the prohormone. Peptides that are not derived from known prohormones are labeled with their FASTA annotation as entered in the planarian transcriptome. Unidentified ions were detected via MALDI MSI but were not characterized in the follow-up HPLC-ESI MS/MS peptidomic analysis. Anterior is to the *top* for each panel. Signal intensity is color coded, with intensity scales provided. Intensity increases from *blue* to *red*.

Ion images of intact planarians show several peptide peaks that are localized in or surrounding the cephalic ganglia (Fig. 2). Among these, peptides from known prohormones, such as secreted peptide prohormone 4 (SPP-4; *m*/*z* 1223.9), secreted peptide prohormone 12 (SPP-12; *m*/*z* 1884.1), and EYE53–1 (*m*/*z* 1525.1) show strong intensity within the ganglia (Fig. 2*A*). Signals localized around the ganglia also include unidentified ions such as *m*/*z* 2791.7, 3344.9, and 3458.7 (Fig. 2*B*). For 3- and 6-day regenerated planarians, structures that correspond to the ganglia were not observed, and most signals that were detected in intact planarians were not present. Fig. 3 shows images for several ions of note at these time points as well as optical images of the regenerating planarians. Two ions, *m*/*z* 3344.9 and 3458.7, which were localized around the cephalic ganglia in intact planarians, were consistently detected in the blastema. Additional ions, such as *m*/*z* 1000.6, were also observed. Although ion images of the 3- and 6-day planarians are generally similar, localization differences can be observed. For example, the distributions of *m*/*z* 3344.9 and 3458.7 shift toward the most anterior edge of the animal at 6 days of regeneration compared with 3 days. In images of the 12-day regenerated planarians, the cephalic ganglia can again be seen, along with most of the other signals detected in intact planarians (Fig. 3*B*).

**FIGURE 4.**
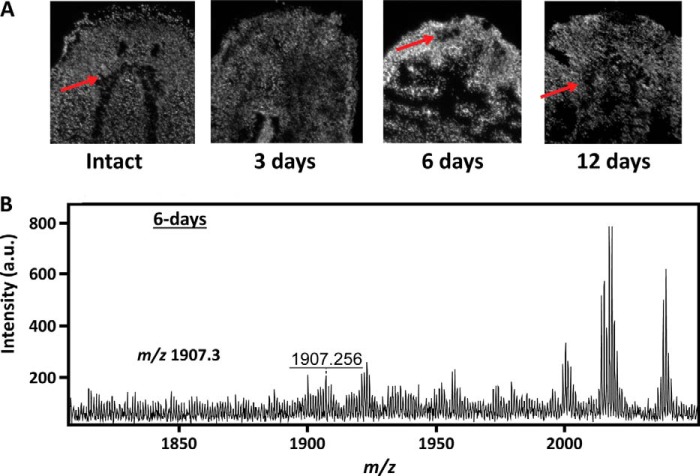
**Validation of the MSI results with DAPI fluorescence imaging.** *A*, DAPI fluorescence images of planarian tissue sections at different regeneration time points. The cephalic ganglia are visible in intact planarians and in planarians at 6 and 12 days of regeneration (*red arrows*). They are not visible in our sample at 3 days of regeneration. *B,* a weak ion signal for the cephalic ganglia at *m*/*z* 1907.3 is observed in 6-day regenerates. Image contrast for the cephalic ganglia is low, likely because of the small ganglia size and low ion intensity.

To validate our MSI results, we conducted DAPI fluorescence imaging as a complementary imaging technique (Fig. 4*A*), which allowed us to visualize the ganglia. Consistent with the ion images, the cephalic ganglia were clearly visible in intact and 12-day regenerated planarians but not in 3-day regenerated planarians. In 6-day regenerated planarians, a structure that resembles the cephalic ganglia was observed. The structure was small and more anterior in the tissue section than its position in intact planarians. Using the DAPI images as a guide, the MSI results from 6-day regenerated planarians were reexamined. A weak signal (near baseline) for an ion at *m*/*z* 1907.3, which is localized to the cephalic ganglia in intact planarians (Fig. 2), was observed in parts of the blastema (Fig. 4*B*), showing that weak ion signal for the cephalic ganglia can be observed at 6 days of regeneration. Taken together, our results and prior studies indicate that although planarian cephalic ganglia begin to appear and express mRNAs at earlier time points, maturation of the cephalic ganglia as monitored by proteomic signatures occurs more slowly.

##### Statistical Analysis Reveals Chemical Trends in Regeneration

In conjunction with analyzing the ion images, PCA was also conducted on the MSI data set to highlight the time course of the chemical changes. To focus the statistical analysis on the regeneration site, for intact and 12-day regenerated planarians, mass spectra from the cephalic ganglia were selected for analysis. For 3- and 6-day regenerated planarians, mass spectra from near the putative cephalic ganglia or the blastema were selected.

**FIGURE 5.**
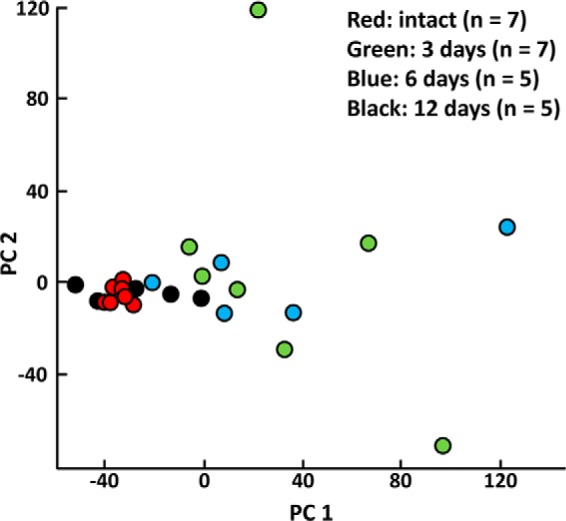
**PCA of intact and regenerating planarians.** Each point is an individual animal. Data points for intact and 12-day regenerates are separated from 3- and 6-day regenerates on principal component 1. Intact and 12-day regenerates also overlap and cluster, whereas the 3- and 6-day regenerates have much greater chemical heterogeneity. This distribution pattern trends with how much time has elapsed since the injury and shows that regeneration is one of the largest factors accounting for chemical variation.

Principal component 1, which accounts for over 30% of the variance, separates intact and 12-day regenerated planarians from 3- and 6-day regenerated planarians (Fig. 5). Data points for intact planarians are clustered together and overlap with data points from 12-day regenerated planarians. Data points for 3- and 6-day regenerated planarians are separated from intact and 12-day planarians, and show more varied distributions on principal components 1 and 2.

Beyond PCA, which is useful for revealing general chemical trends, we also conducted a series of statistical tests on the non-transformed MALDI MS data to determine compounds that varied significantly between regeneration time points. Table 1 lists signals that trended toward higher intensity in the cephalic ganglia. Intensity ratios between regenerating and intact planarians show low intensities at 3 and 6 days of regeneration, and comparable intensities between intact and 12-day regenerated planarians. The predictive power of these compounds for the cephalic ganglia was determined by calculating the area under the receiver operating characteristic curve. Table 2 shows signals that trended toward the blastema. These compounds have opposite trends as those listed in Table 1, becoming more intense at 3 and 6 days of regeneration, before decreasing to close to the intact level at 12 days. Thus, our statistical analyses also indicate that the cephalic ganglia continue to change at the protein level until this later time point during regeneration.

**TABLE 1:** **Ions that are significantly more intense in the cephalic ganglia**

Peptide	Mass	*p* value		Intensity ratio			Relative error			AUC*^c^*
		KW*^a^*	AD*^b^*	3 day/Intact	6 day/Intact	12 day/Intact	3 day/Intact	6 day/Intact	12 day/Intact	
dd_Smed_v4_1463_0_1 (ANTH-like domain)	898.6	4.8 × 10^−2^	2.6 × 10^−2^	4 × 10^−1^	4 × 10^−1^	4 × 10^−1^	1 × 10^−1^	1 × 10^−1^	2 × 10^−1^	0.98
SPP-4 [33–42]*^d^*	1223.9	2.6 × 10^−2^	3.2 × 10^−3^	4 × 10^−1^	3 × 10^−1^	4 × 10^−1^	9 × 10^−2^	6 × 10^−2^	1 × 10^−1^	0.98
EYE53-1 [30–42]	1525.1	2.6 × 10^−2^	1.0 × 10^−6^	5 × 10^−1^	4 × 10^−1^	1 × 10^0^	1 × 10^−1^	6 × 10^−2^	9 × 10^−1^	0.92
SPP-6 [48–62]	1590.0	2.3 × 10^−2^	1.0 × 10^−4^	3 × 10^−1^	4 × 10^−1^	1 × 10^0^	8 × 10^−2^	1 × 10^−1^	4 × 10^−1^	1.00
dd_Smed_v4_15196_0_1 (no SP)*^e^*	1646.0	2.6 × 10^−2^	9.3 × 10^−4^	3 × 10^−1^	3 × 10^−1^	9 × 10^−1^	7 × 10^−2^	6 × 10^−2^	3 × 10^−1^	1.00
SPP-12 [57–70]	1884.1	2.6 × 10^−2^	2.7 × 10^−3^	2 × 10^−1^	3 × 10^−1^	7 × 10^−1^	4 × 10^−2^	9 × 10^−2^	3 × 10^−1^	1.00
Not detected by HPLC-ESI MS/MS	1901.1	3.8 × 10^−2^	1.5 × 10^−2^	4 × 10^−1^	5 × 10^−1^	7 × 10^−1^	6 × 10^−2^	2 × 10^−1^	2 × 10^−1^	1.00
Smed_v4_1178_0_1 (SP)*^e^*	1907.3	2.6 × 10^−2^	1.2 × 10^−3^	2 × 10^−1^	3 × 10^−1^	4 × 10^−1^	7 × 10^−2^	6 × 10^−2^	1 × 10^−1^	1.00
Not detected by HPLC-ESI MS/MS	2274.3	2.6 × 10^−2^	2.1 × 10^−4^	4 × 10^−1^	5 × 10^−1^	1 × 10^0^	1 × 10^−1^	9 × 10^−2^	3 × 10^−1^	0.92

*^a^ p* values calculated via the Kruskal-Wallis analysis of variance.

*^b^ p* values calculated via the Anderson-Darling test for the normal distribution assumption.

*^c^* Area under the receiver operating characteristic curve.

*^d^* Amino acid positions in known prohormones are shown in brackets.

*^e^* Indicates the presence of signal peptide.

**TABLE 2:** **Ions that are significantly more intense in the blastema**

Peptide	Mass	*p* value		Intensity ratio			Relative error			AUC*^c^*
		KW*^a^*	AD*^b^*	3 day/Intact	6 day/intact	12 day/intact	3 day/intact	6 day/intact	12 day/intact	
dd_Smed_v4_5053_0_1 (RNA recognition motif)	864.5	2.3 × 10^−2^	7.1 × 10^−3^	3 × 10^0^	2 × 10^0^	8 × 10^−1^	5 × 10^−1^	2 × 10^−1^	2 × 10^−1^	0.96
dd_Smed_v4_89998_0_1 (Myb-type HTH DNA-binding domain)	871.5	2.3 × 10^−2^	1.0 × 10^−6^	6 × 10^0^	5 × 10^0^	1 × 10^0^	2 × 10^0^	2 × 10^0^	4 × 10^−1^	0.98
Not detected by HPLC-ESI MS/MS	958.6	2.6 × 10^−2^	2.7 × 10^−3^	3 × 10^0^	2 × 10^0^	1 × 10^0^	7 × 10^−1^	4 × 10^−1^	2 × 10^−1^	0.96
Not detected by HPLC-ESI MS/MS	977.6	2.6 × 10^−2^	4.3 × 10^−2^	2 × 10^0^	2 × 10^0^	1 × 10^0^	4 × 10^−1^	4 × 10^−1^	2 × 10^−1^	0.96
dd_Smed_v4_1805_0_1 (UBA-like Domain)	980.6	2.6 × 10^−2^	2.9 × 10^−6^	3 × 10^0^	3 × 10^0^	1 × 10^0^	1 × 10^0^	8 × 10^−1^	2 × 10^−1^	0.86
dd_Smed_v4_918_0_1 (no SP)*^d^*	1000.6	2.3 × 10^−2^	1.0 × 10^−6^	4 × 10^0^	5 × 10^0^	1 × 10^0^	2 × 10^0^	2 × 10^0^	3 × 10^−1^	0.94
dd_Smed_v4_26194_0_1 (Histone H4)	1015.6	2.6 × 10^−2^	2.7 × 10^−3^	2 × 10^0^	3 × 10^0^	1 × 10^0^	4 × 10^−1^	7 × 10^−1^	2 × 10^−1^	0.92
dd_Smed_v4_1350_0_1 (no SP)*^d^*	1136.7	5.0 × 10^−2^	1.6 × 10^−4^	3 × 10^0^	4 × 10^0^	1 × 10^0^	1 × 10^0^	1 × 10^0^	5 × 10^−1^	0.92
Not detected by HPLC-ESI MS/MS	1139.7	3.2 × 10^−2^	4.2 × 10^−4^	3 × 10^0^	2 × 10^0^	1 × 10^0^	6 × 10^−1^	5 × 10^−1^	2 × 10^−1^	0.96
Not detected by HPLC-ESI MS/MS	1285.8	2.3 × 10^−2^	2.2 × 10^−2^	2 × 10^0^	2 × 10^0^	9 × 10^−1^	2 × 10^−1^	3 × 10^−1^	1 × 10^−1^	0.96
dd_Smed_v4_2006_0_1 (Regulator of Vps4 in MVB pathway)	1329.8	3.8 × 10^−2^	3.7 × 10^−5^	2 × 10^0^	2 × 10^0^	2 × 10^0^	3 × 10^−1^	3 × 10^−1^	8 × 10^−1^	0.90
Not detected by HPLC-ESI MS/MS	1348.8	2.3 × 10^−2^	1.7 × 10^−3^	3 × 10^0^	3 × 10^0^	1 × 10^0^	5 × 10^−1^	7 × 10^−1^	3 × 10^−1^	1.00
dd_Smed_v4_10215_0_1 (no SP)*^d^*	1490.9	2.6 × 10^−2^	1.2 × 10^−6^	2 × 10^0^	2 × 10^0^	1 × 10^0^	4 × 10^−1^	6 × 10^−1^	1 × 10^−1^	0.88
Not detected by HPLC-ESI MS/MS	3458.7	2.3 × 10^−2^	8.5 × 10^−4^	6 × 10^0^	5 × 10^0^	3 × 10^0^	1 × 10^0^	2 × 10^0^	4 × 10^−1^	0.90

*^a^ p* values calculated via the Kruskal-Wallis analysis of variance.

*^b^ p* values calculated via the Anderson-Darling test for the normal distribution assumption.

*^c^* Area under the receiver operating characteristic curve.

*^d^* Indicates the presence of signal peptide.

##### Peptidomic Analysis to Identify Regeneration Markers

Peptidomic analysis was conducted to identify the regeneration markers detected via MSI. Database searching was conducted against the Uniprot *S. mediterranea* protein database. Identification results from this effort are presented in Tables 1 and 2, along with information on observed peptide intensities discussed in the previous section. These results are further elaborated in Table 3 with sequences obtained via MS/MS. Sequences that match known prohormones are presented in bold. Peptides that do not match entries in the protein database were also detected. For these sequences, obtained *de novo* peptide sequence tags were searched against the planarian transcriptome and against sequences from proteins in other organisms. The non-bolded sequences in Table 3 are complete sequences obtained in this manner. However, because each sequence contains residues with varying amounts of support from the associated MS/MS spectrum, it is typically difficult to find BLAST results that match the entire *de novo* sequence. The presented BLAST results therefore focus on matching residues with local confidence above 80%, as indicated by the PEAKS software package. Focusing on high-confidence residues reduced the impact on the search results by residues that were weakly supported by MS/MS. For example, sequences containing a single cysteine that is modified with half a disulfide bond are unexpected, as two cysteine residues are required to make a disulfide bond, and these samples were not treated to reduce disulfide bonds prior to analysis. In these sequences, the modified cysteine residue had a low confidence score and was not focused on for the search. BLAST searching in this manner revealed proteins that are involved in a variety of functions, such as histones, and proteins involved in cellular trafficking, DNA binding, etc. These BLAST matches are presented in Tables 1 and 2, along with their FASTA annotation. Sequences without BLAST matches were also detected and are also listed in Table 3 with their FASTA annotation. One of these contains a characteristic signal peptide near the N terminus, suggesting that it may be destined for the secretory pathway.

**TABLE 3:** **Peptidomic identification of MALDI MSI regeneration markers via MS/MS** Bold entries are those that match known prohormones. Underlined residues are those obtained from *de novo* sequencing with local confidence higher than 80%. Descriptions include known prohormones and BLAST results obtained by searching the *de novo* sequences against the transcriptome, focusing on matching the underlined residues. Those without BLAST results are shown with their FASTA annotation. Putative PTMs include amidation (a), acetylation (ac), oxidation (o), pyroglutamination (p), and half of a disulfide bond (*).

*m/z*	Sequence	Description
**Peptide markers for the cephalic ganglia**		
898.6	FSASRAN-Mo,a	ANTH-like domain
1223.9	**DSYHRYPSSI**	**Secreted peptide prohormone 4**
1525.1	**LSIPTYWDDIDTS**	**EYE53–1**
1590.0	**LIDP-Mo-TFGYGFSNL**	**Secreted peptide prohormone 6**
1646.0	NWEDFFFKDAA-Kac-Sa	dd_Smed_v4_15196_0_1 (no signal peptide)
1884.1	**pQ-QFFRNHRPEIEWN**	**Secreted peptide prohormone 12**
1907.3	PVSMPFKWADYYKFQ	dd_Smed_v4_1178_0_1 (has signal peptide)

**Peptide markers for the blastema**		
864.5	oM-INDP-C*-A-Sa	RNA recognition motif
871.5	TMVYG-C*-Ea	Myb-type HTH DNA-binding domain
980.6	acF-SRDDAL-Da	UBA-like domain
1000.6	ATWRDFFG	dd_Smed_v4_918_0_1 (no signal peptide)
1015.6	STGKGGNAPG-Kac	Histone H4
1136.7	MTVCDHFGI-Da	dd_Smed_v4_1350_0_1 (no signal peptide)
1329.8	acY-GNGPMHLGPD-Ma	Regulator of Vps4 in MVB Pathway
1490.9	E-Kac-d-Mo-VAQPEVAN-Ca	dd_Smed_v4_10215_0_1 (no signal peptide)

##### Global Peptidomic Analysis of Regenerating Planarians

Beyond identifying markers that were determined via MSI, global peptidomic analysis also detected peptides from many known *S. mediterranea* prohormones (supplemental Table S1), such as secreted peptide prohormones (SPP), pedal peptide prohormones (PPP), neuropeptide Y prohormones (NPY), 1020HH, and EYE53. Other than prohormones, we observed peptides from protein fragments, *e.g.* metallopeptidases, clathrin, and histone. Peptides that did not match the protein database were sequenced *de novo*, searched against the planarian transcriptome database, and evaluated for BLAST matches. These tags were filtered to keep only those sequences in which the average local confidence for all residues was higher than 80%, and searches were again focused on matching residues with local confidence over 80%. A summary of these matches is shown in supplemental Table S2. Sequences in the transcriptome database that do not have BLAST matches were also detected. These are described in supplemental Table S2, along with an indication of the presence or absence of a signal peptide.

##### Validating Peptide and Protein Enrichment via In Situ Hybridization

**FIGURE 6.**
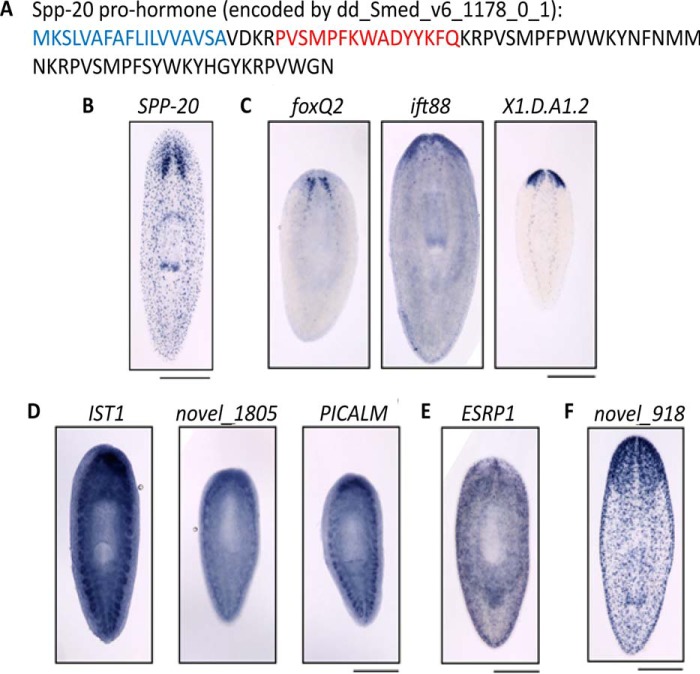
**Transcript localization for candidate genes.** *A,* the up-regulated peptide with *m*/*z* 1907.3 is encoded by a transcript previously identified in transcriptomic analysis (*dd_Smed_v4_1178_0_1* or *dd_Smed_v6_1178_0_1*). The protein translated from this transcript includes a signal peptide (*blue*) and the peptide identified by MS (*red*). Because we expect that this gene product encodes a novel neuropeptide, we have named it *spp-20* (*secreted peptide prohormone-20*). *B*, using *in situ* hybridization, we confirmed that the *spp-20* gene is expressed in cells of the planarian brain, as well as in puncta throughout the planarian body, which could be part of the peripheral nervous system. *C,* the transcripts *foxQ2*, *ift88*, and *X1.D.A1.2* are enriched in the anterior of animals after head amputation, in head or CNS tissues. *D*, *IST1* (dd_Smed_v4_2006_0_1), *novel_1805* (dd_Smed_v4_1805_0_1) and *PICALM* (dd_Smed_v4_1463_0_1) genes are expressed broadly, with enrichment of transcripts in the blastema tissue of regenerating animals. *E*, *ESRP1* (dd_Smed_v4_5053_0_1) showed no such mRNA enrichment in the blastema. *F,* our approach allowed the identification of novel genes expressed in the planarian head and CNS, including *novel_918* (dd_Smed_v4_918_0_1), which shows head enrichment in an uninjured animal. For all experiments, anterior is to the *top* of the page. *Scale bars*, 0.5 mm.

To validate the blastema and cephalic ganglia-enriched proteins identified in this study, we investigated whether the corresponding mRNAs are expressed in these locations. Of the ions detected in the cephalic ganglia, some were translated from mRNAs that we previously showed to be enriched in the planarian CNS, including *spp-4*, *eye53-1*, *spp-6*, and *spp-12* (23). We were interested to see whether novel ions identified by MS were encoded by transcripts similarly expressed in the planarian CNS. We focused on one ion (*m*/*z* 1907.3), which is encoded by a novel gene (Fig. 6*A*). This gene, which we have named *secreted peptide prohormone-20* (*spp-20*) in sequence after the previously identified planarian genes, encodes a predicted neuropeptide prohormone that contains a signal peptide (Fig. 6*A*, *blue*) (53) and can be processed to several smaller peptides, including the peptide identified by MS (Fig. 6*A*, *red*). We used *in situ* hybridization to detect the *spp-20* transcript and found that it was, indeed, enriched in the planarian brain and in puncta throughout the animal that could be part of the peripheral nervous system (Fig. 6*B*). Several peptides were produced by transcripts enriched in the CNS or head, including *foxQ2*, *ift88*, and *X1.D.A1.2* (Fig. 6*C*) (56, 57). Additional peptides encoded by the transcripts *IST1*, *novel_1805,* and PICALM were up-regulated generally throughout the blastema tissue (Fig. 6*D*). However, there were occasions in which a peptide was enriched without clear up-regulation of the corresponding mRNA, such as *epithelial splicing regulatory protein 1* (*ESRP1* (Fig. 6*E*)). Such protein-specific enrichment could either represent a posttranscriptional event that results in up-regulation of protein without increased expression of the transcript or indicate a false positive due to the MS methodology used. Finally, the MS approach utilized in this study allowed the identification of previously uncharacterized genes and their products, which we can confirm are enriched at the transcript level in the blastema (Fig. 6*D*) and in the CNS (Fig. 6*F*).

## Discussion

Tissue regeneration is a complex process involving the coordination of cell division and differentiation, likely guided by a suite of biochemicals that vary spatially and temporally. MSI is well suited for conducting *de novo* analyses of these complex processes because it is an untargeted and multiplex analytical method. In this study, we applied MALDI MSI to image peptide distribution changes that are associated with cephalic ganglia regeneration in *S. mediterranea*.

### 

#### 

##### Mass Spectrometry Imaging Reveals Chemical and Structural Trends of Regeneration

After head amputation, the cephalic ganglia were not observed via MALDI MSI until after 12 days of regeneration (Fig. 3*B*). Ion images are further supported by our statistical analyses (*e.g.* PCA), which show similarities between intact and 12-day regenerates but not between intact and 3- or 6-day regenerates. Our results are interesting compared with previous studies on planarian anterior regeneration that have reported the formation of small neural structures after just a few days (3, 7, 58). Even with a combination of DAPI fluorescence imaging and MSI, we only observed indistinct ion distributions near the cephalic ganglia in 6-day regenerated planarians. There are several possibilities for this discrepancy. One is that the small ganglia structures at 3 and 6 days of regeneration may be missed by the 50-μm MALDI laser rastering steps. Another potential issue is related to sample preparation. MSI is incompatible with many fixation techniques used for preserving morphology because fixation can negatively affect MS signals (59, 60), thus requiring additional sample preparation protocols (61). Our samples were therefore processed without any fixation treatment. Even though we had taken care to reduce sample damage by embedding planarians in gelatin and incorporating supercritical drying, it is possible that parts of the blastema were lost during sample preparation. Despite these potential limitations, our results still produced robust trends.

Although many studies report that the cephalic ganglia are formed before 12 days of regeneration, chemical differences, such as in neurotransmitter and metabolite content, are often detected (16, 62). This is consistent with our data, which show that even though the ganglia were observed in DAPI fluorescence imaging at 6 days (Fig. 4), the protein signatures remained weak, showing that chemical changes continued to occur after the ganglia were structurally present. In fact, our MSI data show that chemical differences exist even at 12 days of regeneration. For example, a peptide derived from SPP-4, which is expressed in the cephalic ganglia, appears not to have recovered to the intact level at the 12-day time point (Tables 1 and 3). The longer time frame shown via MSI may also suggest that neuropeptide and protein contents are more characteristic of mature neuronal tissues. Neuropeptide production requires multiple processing enzymes to cleave prohormones and to modify the formed peptide (63). It is possible that less mature tissues do not possess the full set of processing enzymes, or certain key enzymes are present at lower abundance, thus hindering neuropeptide production during the early regeneration stages.

##### Peptidomic Analysis of Regenerating Planarians

Our identification workflow relied on comparing MALDI MSI signals against identification results from HPLC-ESI MS/MS. This approach is common in MSI studies and is effective in many cases, but it is unable to identify compounds that preferentially ionize via MALDI over ESI (64). Not all markers detected with MSI could therefore be identified. Our peptidomics analysis also focused on detecting changes in endogenous peptides. Many changes in protein composition were therefore not detected, which is a limitation of our approach. Nonetheless, we detected a variety of peptides derived from prohormones or related to proteins. Although these peptides and proteins are likely present in intact planarians, many may also play a role during regeneration, and so their levels may be changed.

Among prohormones, we detected peptides derived from prohormones of the SPP, PPP, and NPY families. These peptides influence a large number of biological processes. Planarian PPP-derived peptides show sequence similarities to those from mollusks and may be involved in locomotion (65). Peptides from the NPY-8 prohormone are necessary for normal sexual development in the sexual strain of *S. mediterranea* (23). As expected, NPY-8 peptides were not detected in this study because the asexual strain was used. A large number of SPP peptides were detected. Among these are peptides from SPP-6, -7, -8, -9, and -17, which are encoded by paralogous genes and show similar localization in the CNS (23). EYE53 and 1020HH are both expressed in eyespots, the photosensing organs of the planarian (62).

Among detected proteins, metallopeptidases are involved in remodeling the extracellular matrix and have been implicated in previous transcriptomic studies on planarian anterior regeneration (24). Thymosin β-4 is involved in various aspects of cell differentiation and proliferation during wound repair (66). Our peptidomics results are consistent with an organism that is in a healing state, revealing proteins involved in tissue remodeling, protein synthesis, and general wound repair (24, 66).

**FIGURE 7.**
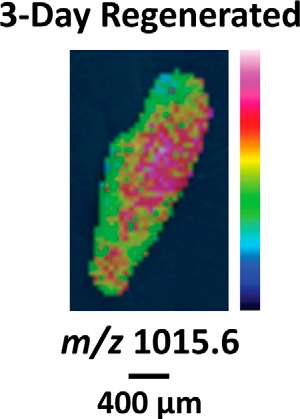
**Ion image for histone H4 in 3-day regenerates.** Signal is detected throughout the body, but it trends toward the base of the blastema and is not detected within the blastema.

One peptide marker detected via MALDI MSI and associated with the blastema is identified as histone H4. It is interesting to observe a trend in histone level because histone is present in similar levels in all cells. Histone synthesis is up-regulated during DNA replication in proliferating cells (67), and histone H4 mRNA expression is restricted to proliferating neoblasts and germ cells in *S. mediterranea* (68). During regeneration, neoblasts proliferate at the base of the blastema, and post-mitotic neoblast progeny enter the blastema during the course of differentiation (28, 69). For a few 3-day regenerates, we observed a stronger histone H4 signal at the base of the blastema compared with inside the blastema (Fig. 7) (70). Perhaps the proliferating neoblasts have a higher content of newly synthesized histones that have not yet been incorporated into chromatin, altering detection via MS.

Beyond known prohormones and proteins, our MS-based approach also enabled the discovery of a new prohormone gene that we have designated as *spp-20*. This gene is novel and does not have a human homolog, but homologous genes do exist in other planarian species that have regenerative capability. Although the function of SPP-20 is currently unknown, our data show that neuropeptides derived from *spp-20* are associated with more mature cephalic ganglia in 12-day regenerates rather than with early tissue regeneration. Further studies will be required to assay the function of *spp-20* and other neuropeptides in planarian regeneration.

In a recent MALDI MS investigation of the planarian anterior regeneration proteome by Chen *et al.* (39), a number of proteins with parallels to the ones we observed were detected. These included structural proteins, proteins involved in the inflammatory response process, and those that participate in transcription and translation. Of note, a few proteins related to cell-cell signaling were the last to be up-regulated or down-regulated during the investigated time period; *e.g.* regulators of neuronal projection, such as tyrosine protein kinase-7, which was up-regulated at 120 h (5 days) after amputation. This pattern is interesting in light of our data showing that many neuropeptides do not return to the intact level until late into cephalic ganglia regeneration. Perhaps the synthesis of specific cell-to-cell signaling molecules is delayed until most neuronal structures and synapses have been formed.

MALDI MSI has also been used to investigate leech regeneration (40, 41). Specific peptides, such as fragments of an intermediate filament protein, showed distinct expression biases near the wound site and indicated structural remodeling associated with regrowth. Multiple proteins involved in neurotransmission, such as sodium channels, were also among the last to be reestablished, consistent with what is observed in planarian regeneration, and suggests similarities in nervous system regeneration across model organisms. These other MSI analyses were extended beyond peptides and proteins to include lipid changes, specifically focusing on the role of cannabinoids. Similar MSI investigations into lipid distribution may represent an interesting new direction for future analyses of regeneration in planarians and other models.

## Author Contributions

T. H. O. designed the study, optimized the MSI experiments, performed many of the experiments, analyzed the data, and wrote the paper. E. V. R. designed the study, provided advice on the experiments, analyzed the data and wrote the paper. R. H. R-G. performed animal culture, optical microscopy, *in situ* experiments and wrote the paper. N. Y. performed many of the FTMS measurements and database searches. T. A. Z. performed some of the initial experiments on planarian MSI. J. E. L. performed the initial FTMS measurements. J. J. C. aided in experimental design and data analysis, and edited the paper. N. L. K. aided the experimental design and analysis of the FTMS data. P. A. N. aided experimental design of the *in situ* and optical microscopy experiments, data analysis, and edited the paper. J. V. S. conceived and designed the study, analyzed the data, and wrote the paper. All authors approved the final version of the manuscript.

## Supplementary Material

Supplemental Data

## Footnotes

6 The abbreviations used are:

MSI: mass spectrometry imaging
PCA: principal component analysis
FA: formic acid
ACN: acetonitrile
ESI: electrospray ionization
QTOF: quadrupole TOF
ICR: ion cyclotron resonance
FT: Fourier transform
SPP: secreted peptide prohormones
PPP: pedal peptide prohormones
NPY: neuropeptide Y prohormones.
