# Supplementary material for: Mass Spectrometry Imaging and Identification of Peptides Associated with Cephalic Ganglia Regeneration in Schmidtea mediterranea
Source: J Biol Chem. 2016 Feb 16;291(15):8109–20. doi: 10.1074/jbc.M115.709196 (PMC4825013; doi:10.1074/jbc.M115.709196)
Supplement: Supplemental Data [file supp_291_15_8109__index.html]

Mass Spectrometry Imaging and Identification of Peptides Associated with Cephalic Ganglia Regeneration in Schmidtea mediterranea — Mass Spectrometry Imaging and Identification of Peptides Associated with Cephalic Ganglia Regeneration in Schmidtea mediterranea — Mass Spectrometry Imaging of Peptides in Tissue Regeneration — Supplemental Data 

# Mass Spectrometry Imaging and Identification of Peptides Associated with Cephalic Ganglia Regeneration in *Schmidtea mediterranea*

## Supplemental Data

- Supplementary Tables 1 and 2 (.pdf, 154 KB) - Supplementary Tables
