# Supplementary material for: Mass Spectrometry Imaging and Identification of Peptides Associated with Cephalic Ganglia Regeneration in Schmidtea mediterranea
Source: J Biol Chem. 2016 Feb 16;291(15):8109–20. doi: 10.1074/jbc.M115.709196 (PMC4825013; doi:10.1074/jbc.M115.709196)
Supplement: Supplemental Data [file 10.1074_M115.709196_jbc.M115.709196-1.pdf]

Mass Spectrometry Imaging and Identification of Peptides Associated with Cephalic Ganglia Regeneration in *Schmidtea mediterranea*

Ta-Hsuan Ong[1], Elena V. Romanova[1], Ning Yang[1], Tyler A. Zimmerman[1^], James J. Collins[2,%], Rachel Roberts-Galbraith[2], Ji Eun Lee[1,#], Neil Kelleher[3], Phillip A. Newmark[2], Jonathan V. Sweedler[1,]*

[1]Department of Chemistry, and the Beckman Institute, University of Illinois at Urbana-Champaign, Urbana, Illinois 61801, United States
[2]Department of Cell and Developmental Biology, University of Illinois at Urbana-Champaign, Urbana, Illinois 61801, United States
[3]Department of Chemistry, Department of Molecular Biosciences, Proteomics Center of Excellence, Northwestern University, Evanston, IL, USA

Current addresses:
[^]Mass Spectrometry Data Center Group, National Institute of Standards and Technology, Gaithersburg, MD 20899, United States
[%] Department of Pharmacology, University of Texas Southwestern Medical Center, Dallas, TX 75390, USA
[#]Center for Theragnosis, Biomedical Research Institute, Korea Institute of Science and Technology, Seoul 136-791, Republic of Korea

SUPPLEMENTAL DATA

Table S1. Identified peptides from HPLC–ESI-(Q)TOF MS/MS and HPLC-ESI-FT-ICR MS/MS conducted on regenerating *S. mediterranea*. Peptides that were identified using PEAKS Studio are shown with their associated score percent and score (-10lgP). Peptides identified using ProSight PC are shown with their associated E-value.

Accession	Protein/Prohormone	Sequence	PEAKS		ProSight
			Score (%)	-10lgP	E-Value
V9XRL6|V9XRL6_SCHMD	1.C5.1	VQNYELE-Mo-PEIIA	57.9	24.2	-
E3T7U2|E3T7U2_SCHMD	1020HH-1 protein	YLSDGTSLENFDDLESNSDY-Mo	96.2	33.96	-
E3T7U2|E3T7U2_SCHMD	1020HH-1 protein	YLSDGTSLENFDDLESNSDY-Mo,a	89.2	30.58	-
E3CTK0|E3CTK0_SCHMD	1020HH-2	VPRSAENLRTYDSLLEELNDYDPIY	99.8	49.25	-
H2DL21|H2DL21_SCHMD	Caspase 2	IIFSCCRGETAALASMLVMESISHSDSLSINDEIQKDRYDEFAWQVGNYIP	-	-	8.56E-07
I1ZIF8|I1ZIF8_SCHMD	Clathrin heavy chain-1	AGPMGIPN-Mo-YGAGSVGPGFA	96	32.11	-
I1ZIF8|I1ZIF8_SCHMD	Clathrin heavy chain-1	LTAGP-Mo-GIPN-Mo-YGAGSVGPGFA	96	32.43	-
R4PLE1|R4PLE1_SCHMD	Dmd-3 (Fragment)	DSISSLNNQLTS	-	-	1.36E-06
E3CTK8|E3CTK8_SCHMD	EYE53-1	LSIPTYW	93.1	30.51	-
E3CTK8|E3CTK8_SCHMD	EYE53-1	LSVPTYFDDWES	99.9	75.9	-
E3CTK8|E3CTK8_SCHMD	EYE53-1	LSIPTYWDDIDTS	99.9	79.81	-
E3CTJ9|E3CTJ9_SCHMD	EYE53-2	AVVPDAWDDWEI	99.9	72.45	-
J7FNB1|J7FNB1_SCHMD	FoxQ2 (Fragment)	PYFWPHNIEDEDK	-	-	1.03E-09
T1DF42|T1DF42_SCHMD	Fzd-5/8-3	NLLNEVSYQLT	-	-	2.18E-05
I1ZI85|I1ZI85_SCHMD	GLI pathogenesis related-1 (Fragment)	SASKAQITADKCIFAHDT	-	-	1.30E-5

Q1WC09|Q1WC09_SCHMD	Glycogen synthase kinase 3	LLEYTPSTRVSPIESCTHSFFDELRQESTKLPNDKPLPPLFNFASNEI	-	-	5.41E-05
S5VL05|S5VL05_SCHMD	Histone H1 (Fragment)	KAKATPKKVAPKKTPKKAPVKAKK	99.4	55.56	-
S5W362|S5W362_SCHMD	Histone H2A (Fragment)	AILLPKKTPKESS	70.7	27.37	-
S5W362|S5W362_SCHMD	Histone H2A (Fragment)	GVTIAQGGVLPNIQAILLPKKTPKESS	96.6	36.85	-
S5W362|S5W362_SCHMD	Histone H2A (Fragment)	IAQGGVLPNIQAILLPKKTPKESS	99.9	59.1	-
S5VQT0|S5VQT0_SCHMD	Histone H3 (Fragment)	ARTKQTAR-Kac-STGG-Kac-APRKQ	56	21.89	-
V5SM21|V5SM21_SCHMD	Insulin gene enhancer protein isl-1	ILDDSSMNSDIDDI	-	-	2.51E-06
A0A060Q785|A0A060Q785_SCHMD	Metalloendopeptidase	acS-GQ-C*-Ga	76	30.31	-
A0A060Q785|A0A060Q785_SCHMD	Metalloendopeptidase	SVDSMKMGD-Mo-LL	99.4	41.77	-
I1ZIC3|I1ZIC3_SCHMD	Metalloendopeptidase	ATENLHVGD-Mo-LL	98.4	34.59	-
K0JBS3|K0JBS3_SCHMD	Mmp1 protein	EFYDYDKSTGSLK	99.9	73.05	-
K0JBS3|K0JBS3_SCHMD	Mmp1 protein	FKSSEEKDAEKYLT	97.2	37.56	-
K0JBS3|K0JBS3_SCHMD	Mmp1 protein	GAKFPPS	43	22.39	-
K0JBS3|K0JBS3_SCHMD	Mmp1 protein	DSLKDLQKMAGIEADGKLS	99.2	47.29	-
K0JBS3|K0JBS3_SCHMD	Mmp1 protein	FKSSEEKDAEKYLTEFYDYDKSTGSLKDSLK	99.9	83.53	-
K0JBI8|K0JBI8_SCHMD	Mmp2 protein	FESSDEKSVEKFLS	97.7	47.65	-
K0JBI8|K0JBI8_SCHMD	Mmp2 protein	DYNEKSDKLSDAVKNIQK-Mo-AGLPTDGKITKELLA	99.1	43.7	-
Q49BF9|Q49BF9_SCHMD	Netrin 1	EITSTCGQNKKQWICV	-	-	3.16E-07
E3T7U4|E3T7U4_SCHMD	Neuropeptide 12	RYFQPQQDFPKSNIFQLPESEFENDDVIQ	75.2	27.77	-
E3T7U4|E3T7U4_SCHMD	Neuropeptide 12	pQ-PQQDFPKSNIFQLPESEFENDDVIQ	95.1	35.07	-

E3T7V1|E3T7V1_SCHMD	Neuropeptide 8	DPRFSDQVWHSG-Ya	11.4	26.72	-
E3CTJ4|E3CTJ4_SCHMD	Neuropeptide Y prohormone-2	SANPLKW-Mo-TL	98.5	36.67	-
E3CTI4|E3CTI4_SCHMD	Neuropeptide Y prohormone-3	NKDELDILF	99.6	44.17	-
E3CTL4|E3CTL4_SCHMD	Neuropeptide Y prohormone-4	QTNWYDMKNYEGNENYD S	59.3	26.44	-
E3CTL5|E3CTL5_SCHMD	Neuropeptide Y prohormone-9	LTKQKYSLFSGPEDLRNYL RQLNEYIALSSRPR-Ya	99.6	45.19	-
E3CTK2|E3CTK2_SCHMD	Neuropeptide-18	RNMDLDEYDSLLPKD	18.7	28.33	-
E3CTK2|E3CTK2_SCHMD	Neuropeptide-18	GAEFFIRRV-Va	9.1	25.04	-
E3CTK2|E3CTK2_SCHMD	Neuropeptide-18<br>Secreted peptide prohormone-10	GAEFFI/LR	40.6	29.52	-
E3CTL0|E3CTL0_SCHMD	Neuropeptide-2	pQ-LLNSKQSIFQDEY	99.9	54.03	-
E3CTL0|E3CTL0_SCHMD	Neuropeptide-2	NSKQSIFQDEY	32.5	23.55	-
E3CTL0|E3CTL0_SCHMD	Neuropeptide-2	pQ-LLNSKQSIFQDEY	22.7	22.67	-
E3CTJ0|E3CTJ0_SCHMD	Neuropeptide-5	LNNPFEKSLILN	73.7	22.13	-
E3CTJ0|E3CTJ0_SCHMD	Neuropeptide-5	LNNPFEKSLILNNSEFED	99.9	62.79	-
E3CTL6|E3CTL6_SCHMD	Pedal peptide prohormone like-1	FYNDPLGVALLK	99.2	45.95	-
E3CTL6|E3CTL6_SCHMD	Pedal peptide prohormone like-1	FYNDPLGVALLKSRFDKDS IN	98.5	35.42	-
E3CTL6|E3CTL6_SCHMD	Pedal peptide prohormone like-1	GAYYDPIGGGLL	99.9	56.92	-
E3CTL6|E3CTL6_SCHMD	Pedal peptide prohormone like-1	RSFYDPIGGGLL	99.9	48.67	-
E3CTL6|E3CTL6_SCHMD	Pedal peptide prohormone like-1	SSYYDPIGGGLL	99.7	44.2	-
E3CTL6|E3CTL6_SCHMD	Pedal peptide prohormone like-1	VALLKSRFDKDSIN	80.7	38.34	-
E3CTL6|E3CTL6_SCHMD	Pedal peptide prohormone like-1	YSYYDSIGSGLL	95.9	32.35	-
E3CTL6|E3CTL6_SCHMD	Pedal peptide prohormone like-1	DSNYDPIGGGLL	99.9	57.11	-
E3CTL6|E3CTL6_SCHMD	Pedal peptide prohormone like-1	SSYYDPIGGGLL	99.9	58.99	-
E3CTL6|E3CTL6_SCHMD	Pedal peptide prohormone like-1	YSYYDSIGSGLL	99.9	63.63	-
E3CTL6|E3CTL6_SCHMD	Pedal peptide prohormone like-1	GAYYDPIGGGLL	99.9	72.72	-
E3CTL6|E3CTL6_SCHMD	Pedal peptide prohormone like-1	RSFYDPIGGGLL	99.9	75	-
E3CTJ3|E3CTJ3_SCHMD	Pedal peptide prohormone like-2	SYMDKIGSDLL	99.4	46.35	-
E3CTJ3|E3CTJ3_SCHMD	Pedal peptide prohormone like-2	FRYFDKIGSDLL	95.6	35.22	-

E3CTJ3|E3CTJ3_SCHMD	Pedal peptide prohormone like-2	SYFDKIGNDLL	99.9	73.17	-
C3U5B2|C3U5B2_SCHMD	Protein Wnt	SGTQEQAVVHAFSSASLLF EIARRCAQNKMAHCSCGT SNPTANQEITQEKLLF	-	-	2.24E-06
E3CTI2|E3CTI2_SCHMD	Pyrokinin prohormone like-1	KNFIYIPDDSFINE-Mo-DRA ID	95.9	32.18	-
E3T7T8|E3T7T8_SCHMD	Secreted peptide prohormone 1	ADIDLDDSRLD	28.7	22.17	-
E3T7T8|E3T7T8_SCHMD	Secreted peptide prohormone 1	EDLGSLNADIDLDDSRLD	100	138.6	-
E3CTJ7|E3CTJ7_SCHMD	Secreted peptide prohormone-3	SVMDDLSKDETYLS	99.7	51.8	-
E3T7U1|E3T7U1_SCHMD	Secreted peptide prohormone 4	DSYHRYPSSI	96.9	36.49	-
E3T7U1|E3T7U1_SCHMD	Secreted peptide prohormone 4	SLNDDSELEDSYHRYPSSI	34.9	24.45	-
E3T7U1|E3T7U1_SCHMD	Secreted peptide prohormone 4	NMNDEFQFRDL	99.4	47.86	-
E3T7U1|E3T7U1_SCHMD	Secreted peptide prohormone 4	DYSSLNDDSELEDSYHRYP SSI	88	34.72	-
E3T7U1|E3T7U1_SCHMD	Secreted peptide prohormone 4	GLRLMR-La	34.1	20.05	-
E3T7V0|E3T7V0_SCHMD	Secreted peptide prohormone 5	NDLFRLLD	99.8	49.87	-
E3T7T9|E3T7T9_SCHMD	Secreted peptide prohormone 6	LIDPMTFGYGFSNL	99.6	43.25	-
E3T7T9|E3T7T9_SCHMD	Secreted peptide prohormone 6	IPGIGFNRNFAIY	99.9	74.27	-
E3T7T9|E3T7T9_SCHMD	Secreted peptide prohormone 6	LIDPMTFGYGFSNL	99.9	77.34	-
E3T7U0|E3T7U0_SCHMD	Secreted peptide prohormone 7	RNLHLY	43.2	22.72	-
E3T7U0|E3T7U0_SCHMD	Secreted peptide prohormone 7	TVGFGFNRNLHLY	99.9	56.34	-
E3T7U3|E3T7U3_SCHMD	Secreted peptide prohormone 8	DP-Mo-TFGSGFANL	98.8	39.13	-
E3T7U3|E3T7U3_SCHMD	Secreted peptide prohormone 8	GFGFNRNMLLY	96.1	32.95	-
E3T7U3|E3T7U3_SCHMD	Secreted peptide prohormone 8	LIDP-Mo-TFG	96	32.49	-
E3T7U3|E3T7U3_SCHMD	Secreted peptide prohormone 8	LIDPMTFGSGFANL	98.9	38.29	-
E3T7U3|E3T7U3_SCHMD	Secreted peptide prohormone 8	LIDPMTFGSGFAN-La	10	22.78	-
E3T7U3|E3T7U3_SCHMD	Secreted peptide prohormone 8	T-Mo-GFGFNRN-Mo-LLY	99.9	51.8	-
E3T7U3|E3T7U3_SCHMD	Secreted peptide prohormone 8	TMGFGFNRNMLLY	99.9	54.29	-
E3T7U3|E3T7U3_SCHMD	Secreted peptide prohormone 8	LIDPMTFGSGFANL	99.9	86.6	-
E3CTK1|E3CTK1_SCHMD	Secreted peptide prohormone-9	LIDPLTFG	98.4	35.68	-
E3CTK1|E3CTK1_SCHMD	Secreted peptide prohormone-9	LIDPLTFGSGFSNL	99.9	46.9	-

E3CTK1|E3CTK1_SCHMD	Secreted peptide prohormone-9	SLPYNPEYELY	94.4	29.72	-
E3CTK1|E3CTK1_SCHMD	Secreted peptide prohormone-9	LIDPLTFGSGFSNL	99.9	90.13	-
E3CTK3|E3CTK3_SCHMD	Secreted peptide prohormone-10	GAEFFLQRV-Ea	71.1	31.36	-
E3CTI9|E3CTI9_SCHMD	Secreted peptide prohormone-11	HQQLFPN	65.3	20.02	-
E3CTI9|E3CTI9_SCHMD	Secreted peptide prohormone-11	NDLLDSEFLNDMNENLE	99.9	58.68	-
E3CTJ8|E3CTJ8_SCHMD	Secreted peptide prohormone-12	pQ-QFFRNHRPEIEWN	1.5	15.72	-
E3CTJ8|E3CTJ8_SCHMD	Secreted peptide prohormone-12	NY-Mo-DFFGLNGD-Mo-QR F	99.9	52.92	-
E3CTJ8|E3CTJ8_SCHMD	Secreted peptide prohormone-12	NY-Mo-DFFGLNGDMQR-Fa	19.5	22.56	-
E3CTJ8|E3CTJ8_SCHMD	Secreted peptide prohormone-12	NYMDFFGLNGDMQRF	99.9	88.02	-
E3CTI8|E3CTI8_SCHMD	Secreted peptide prohormone-15	pQ-SDRGYYPILFD	99	40.21	-
E3CTI8|E3CTI8_SCHMD	Secreted peptide prohormone-15	YPQTYELNQSDRGYYPILF D	99.6	44.76	-
E3CTI8|E3CTI8_SCHMD	Secreted peptide prohormone-15	FDPIQFG	99.9	33.25	-
E3CTL7|E3CTL7_SCHMD	Secreted peptide prohormone-16	pQ-FDPIMYGKLRQ	44	24.77	-
E3CTL7|E3CTL7_SCHMD	Secreted peptide prohormone-16	pQ-FDPIMY-Kac-RQ	75.3	23.3	-
E3CTL7|E3CTL7_SCHMD	Secreted peptide prohormone-16	pQ-SNPYFLSDIRSI	98.5	36.57	-
E3CTL7|E3CTL7_SCHMD	Secreted peptide prohormone-16	pQ-FDPIMY	98.5	44.21	-
E3CTL7|E3CTL7_SCHMD	Secreted peptide prohormone-16	QSNPYFLSDIRSI	99.9	56.45	-
E3CTL7|E3CTL7_SCHMD	Secreted peptide prohormone-16	pQ-FDPIMYGKLRQFY	99.9	65.37	-
E3T7U5|E3T7U5_SCHMD	Secreted peptide prohormone 17	IIDP-Mo-TYGTGFSNL	99.9	55.84	-
E3T7U5|E3T7U5_SCHMD	Secreted peptide prohormone 17	YIQDPDENDTLR	81.1	29.66	-
E3T7U5|E3T7U5_SCHMD	Secreted peptide prohormone 17	YIQDPDENDTLRDFYLQ	91.1	27.51	-
E3T7U5|E3T7U5_SCHMD	Secreted peptide prohormone 17	IIDPMTYGTGFSNL	99.9	71.12	-
E3T7U6|E3T7U6_SCHMD	Secreted peptide prohormone 18	GYHFFRL	69.2	34.03	-
E3T7U8|E3T7U8_SCHMD	Secreted peptide prohormone 19	KHIGHQIFRL	78.8	31.9	-
D4P939|D4P939_SCHMD	Smed-NDK-3 (Fragment)	DPASRVKHQVQYPCFVRE QKEATFRMAKGLSVEFD	-	-	1.13E-07
H9XVZ7|H9XVZ7_SCHMD	SMG-1	QMINILELCICDSSSSDEFIV LSGKLMMSLLHLNIRRGD AAIVDFWCRIFDKMTNLQI	-	-	2.66E-08

I1ZIE8|I1ZIE8_SCHMD	Uncharacterized protein (Fragment)	DALHLAGGIVGGAANLAGGVVD	57.4	23.81	-
I1ZIE8|I1ZIE8_SCHMD	Uncharacterized protein (Fragment)	GAANLAGGVVDGAANLAGAAIDVVAGGPERDRHRSRND	99.9	51.17	-
I1ZIE8|I1ZIE8_SCHMD	Uncharacterized protein (Fragment)	LAGGIVGGAADLAGGVVDGAANLA	99.9	72.02	-
I1ZIE8|I1ZIE8_SCHMD	Uncharacterized protein (Fragment)	WFVDDALH	99.7	50.47	-
I1ZIE8|I1ZIE8_SCHMD	Uncharacterized protein (Fragment)	WFVDDALHLAGGIVGGAA	99.9	55.52	-
L0H970|L0H970_SCHMD	Utx (Fragment)	SYLRTVLDEK	-	-	3.40E-06
I1ZID1|I1ZID1_SCHMD	Vault protein inter alpha trypsin-1 (Fragment)	EPLVTNIEEEFP-Ia	66.1	23.08	-
L0H9P2|L0H9P2_SCHMD	Wdr82-2 (Fragment)	TPNSTSQPINCVAFNPKF	-	-	2.53E-05
V9XMQ5|V9XMQ5_SCHMD	X1.B2.2	GAAWQDML-Wa	95.6	35.11	-
V9XMT1|V9XMT1_SCHMD	X1.D.A1.2	SEQADENQLIDNTG-Pa	99.8	59.1	-
V9XMT1|V9XMT1_SCHMD	X1.D.A1.2	SFDHEFLGGNLRF	98.5	36.85	-
V9XMT1|V9XMT1_SCHMD	X1.D.A1.2	TASFDHEFLGGNLRF	99.2	37.82	-
V9XMT1|V9XMT1_SCHMD	X1.D.A1.2	PLQNPSTTDQFWKDFELAYRRLKDHLEKKNA	99.8	46.81	-
V9XMT1|V9XMT1_SCHMD	X1.D.A1.2	TASFDHEFLGGNLRF	99.9	67.87	-

Putative PTMs include amidation (a), acetylation (ac), oxidation (O), pyroglutamination (p), and half of a disulfide bond (*).

Table S2. *De novo* peptide sequence tags from HPLC–ESI-(Q)TOF MS/MS and HPLC-ESI-FT-ICR MS/MS conducted on regenerating *S. mediterranea*. Sequences are searched against the *S. mediterranea* transcriptome. Underlined residues are those with local confidence higher than 80%. Description includes BLAST matches, and whether or not a signal peptide is present in peptides without matches.

Peptide	ALC (%)	FASTA Descriptor	Description
VAPEEHPVLLT	94	dd_Smed_v4_246_0_5	Actin
acT-SEEEFQTMADQMKNLK	98	dd_Smed_v4_160_0_1	Acyl-CoA-binding protein (ACBP)
MLNELYDCENL-Ma	85	dd_Smed_v4_5383_0_1	Cadherin repeats
HSHHAAAYTLF	86	dd_Smed_v4_8673_0_1	Calpain family cysteine protease
acL-LADDV-Mo-TA	82	dd_Smed_v4_4962_0_2	Cell morphogenesis N-terminal
TAFDKAGGANYGSAK	93	dd_Smed_v4_63_0_1	Cofilin/tropomyosin-type actin-binding protein
GYDFFRL	94	dd_Smed_v4_2463_0_1	dDENN domain
DSLKMPEE	90	dd_Smed_v4_24408_0_4	DNA helicase
HLVDETTHLLK	82	dd_Smed_v4_6941_0_2	Dynein heavy chain
acR-PLLRL	88	dd_Smed_v4_4747_0_1	Elongation factor G, domain IV
LWPSYW-Mo-PWESQ	86	dd_Smed_v4_7988_0_1	F5/8 type C domain
LPAVGIFCAGHIETE-Kac-V	85	dd_Smed_v4_12290_0_1	HCO3- transporter family
MDFLNNRS-Mo-P-Sa	86	dd_Smed_v4_6361_0_1	Histidine phosphatase superfamily
FEPMKLSDMLCPALP-Aa	88	dd_Smed_v4_16523_0_1	Ion transport protein N-terminal
RPPGFSPFR	95	dd_Smed_v4_9725_0_1	Ku70/Ku80 beta-barrel domain
EDELDKVD	89	dd_Smed_v4_2472_0_1	Membrane-bound O-acyltransferase family
L-Mo-SDKDD-Mo-LN-Ra	85	dd_Smed_v4_6344_0_1	Myosin-binding striated muscle assembly
SVLDDLSKDETYLS	84	dd_Smed_v4_6687_0_1	Phosphotyrosine interaction domain
SNFDSFEAGYQ	86	dd_Smed_v4_6480_0_1	Protein kinase domain
acM-EEPKHDSEHQVT	94	dd_Smed_v4_13372_0_1	Rhodopsin-like RPCR protein
AVAGVTKDDLLLK	95	dd_Smed_v4_172_0_1	Ribosomal protein L24e

o,acM-DTSDFKLF	98	dd_Smed_v4_295_0_1	Ribosomal protein S7p/S5e
acA-SFIAKQLVGKQLD	91	dd_Smed_v4_4479_1_1	Synaphin
acH-HVDDALH	88	dd_Smed_v4_64731_0_1	TCP-1/cpn60 chaperonin family
EDEVKIDVNELGSFNKESLK	98	dd_Smed_v4_12_0_1	Thymosin beta-4 family
LPSQLDLEEEKNN	98	dd_Smed_v4_17_0_1	Thymosin beta-4 family
EEQTETNL-Ea	99	dd_Smed_v4_17_0_1	Thymosin beta-4 family
NFGSNVDDWRLYA	95	dd_Smed_v4_9302_0_1	Transcription initiation factor TFIID
o,acM-IDEATKKTLAAILPLK	86	dd_Smed_v4_4590_0_1	Ubiquitin-fold modifier-conjugating enzyme 1
YSDDNLDRFAWRL	92	dd_Smed_v4_2652_0_1	Vacuolar protein sorting-associated protein 35
VNLENDLLRSLLD	92	dd_Smed_v4_8872_0_4	Zinc finger domains
DEFEMDDVIED	85	dd_Smed_v4_1066_0_1	Has signal peptide
YDIDQLNYGAGFTNV	84	dd_Smed_v4_11230_0_1	Has signal peptide
KTLFDKDDEATLKELDNLEPK	98	dd_Smed_v4_18_0_1	Has signal peptide
TVKDSPEFFLL	89	dd_Smed_v4_2014_0_1	Has signal peptide
YDTDHDVFRL	97	dd_Smed_v4_2574_0_1	Has signal peptide
WYPVKEYNNLVEL	94	dd_Smed_v4_2910_0_1	Has signal peptide
TYYLDAIDDGRLL	97	dd_Smed_v4_3151_0_1	Has signal peptide
LLDPMTFGYGFSY-Sa	90	dd_Smed_v4_3845_0_1	Has signal peptide
LIDPLTFGSPESN-Na	88	dd_Smed_v4_3845_0_1	Has signal peptide
acG-YSGSDIIDFPE	87	dd_Smed_v4_561_0_1	Has signal peptide
FANANDGVVPQT-Mo,a	85	dd_Smed_v4_6984_0_1	Has signal peptide
DMNDEFQFRDL	92	dd_Smed_v4_788_0_1	Has signal peptide
PTEPHYPQSGL-Kac	88	dd_Smed_v4_851_0_1	Has signal peptide
APEDQMFDT-Kac-NQ	88	dd_Smed_v4_918_0_1	Has signal peptide
ATWRDFFG	84	dd_Smed_v4_918_0_1	Has signal peptide
acA-Kac-IPFSWWHNL	94	dd_Smed_v4_9673_0_1	Has signal peptide
FDNNDEYDSLLPKD	83	dd_Smed_v4_1117_0_1	No signal peptide
PVSMPFKWADYYKFQ	96	dd_Smed_v4_1178_0_1	No signal peptide

RGLPPT-Kac-LR-Fa	92	dd_Smed_v4_13322_0_1	No signal peptide
WLAKSGEHVMVFD	87	dd_Smed_v4_15396_0_1	No signal peptide
oM-NDDFFKNLRDE	93	dd_Smed_v4_2177_0_1	No signal peptide
KENPVVTGNNQLK	90	dd_Smed_v4_2246_0_1	No signal peptide
ATHSKT-C*-TAPLK	85	dd_Smed_v4_30210_0_1	No signal peptide
LSKDLYFNKYKI	98	dd_Smed_v4_3306_0_1	No signal peptide
NSFSRNNL-Mo-DL	98	dd_Smed_v4_47572_0_1	No signal peptide
pQ-RLLPTQDQYETEPFLQVPLVKGRQF	87	dd_Smed_v4_6313_0_4	No signal peptide
DRYVLHPFHL	85	dd_Smed_v4_88260_0_1	No signal peptide

Putative PTMs include amidation (a), acetylation (ac), oxidation (O), pyroglutamination (p), and half of a disulfide bond (*).
